# Exploration of the Protection of Riboflavin Laurate on Oral Mucositis Induced by Chemotherapy or Radiotherapy at the Cellular Level: What Is the Leading Contributor?

**DOI:** 10.3390/ijms14034722

**Published:** 2013-02-27

**Authors:** Zixue Xuan, Yinghong An, Dexuan Yang, Shanshan Wang, Qishou Xu, Shoujun Yuan

**Affiliations:** 1 School of Graduate Studies, Anhui Medical University, Hefei 230032, Anhui, China; E-Mail: xuanzixue0222@163.com (Z.X.); 2 Department of Pharmacology and Toxicology, Beijing Institute of Radiation Medicine, Beijing 100850, China; E-Mails: rainning_sky@163.com (Y.A.); dexuanyang@163.com (D.Y.); shanshanwang1215@163.com (S.W.); xuqsh517@yahoo.com.cn (Q.X.)

**Keywords:** riboflavin laurate, oral or gastrointestinal mucositis, chemotherapy or radiotherapy, Caco-2 cell monolayer

## Abstract

Oral or gastrointestinal mucositis is a frequent phenomenon in cancer patients receiving chemotherapy or radiotherapy. In addition, several clinical investigations have demonstrated in recent years that riboflavin laurate has the potential to protect the patients from the disease induced by chemotherapy or radiotherapy. In our studies, it is observed that riboflavin laurate can ameliorate either chemotherapy- or radiotherapy-induced toxicities on Helf cells, and the effect is greater than that of riboflavin. In addition, riboflavin laurate is able to transport through the Caco-2 cell monolayer as the prototype, indicating the protective effects may be produced by the prototype of riboflavin laurate, rather than simply by the released riboflavin.

## 1. Introduction

**Figure 1:**
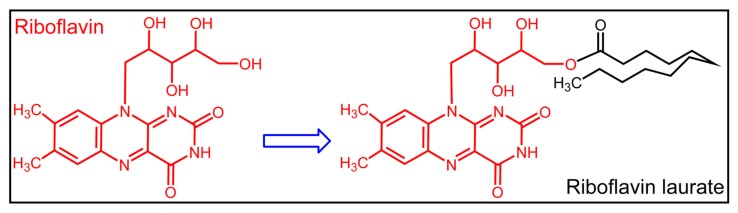
The chemical structures of riboflavin and riboflavin laurate.

Riboflavin (RF) or vitamin B2 (Figure 1) is indispensable and important for normal cellular functions, growth and development in all aerobic forms of life [1]. Flavin adenine mononucleotide (FMN) and Flavin adenine dinucleotide (FAD) are the two major precursors, participating in a myriad of biochemical reactions, including carbohydrate, lipid and amino acid metabolism [2–4]. However, humans are not able to synthesize riboflavin by themselves, and must incorporate it as a micronutrient from their diet [5], so they are vulnerable to develop riboflavin deficiency, especially susceptible are those who work in the wild for a long time, such as soldiers and explorers. Nevertheless, riboflavin deficiency may lead to a variety of clinical abnormalities, like skin inflammation, oral mucositis and scrotitis [6,7].

**Figure 2:**
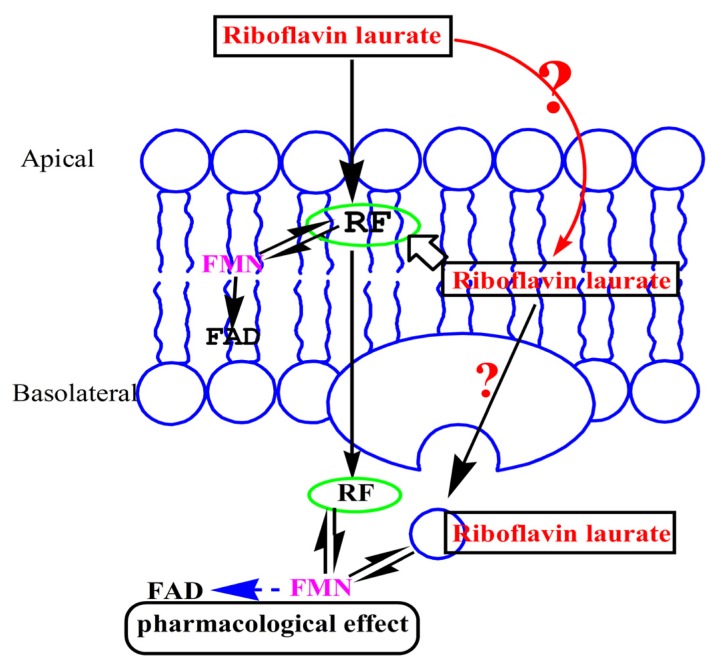
How does the riboflavin laurate transport across the Caco-2 cell monolayer? Current views merely consider that riboflavin, not the free riboflavin laurate, is released to the body over time, when it is injected into the muscle. Caco-2 model is established to demonstrate the transport mechanisms, illuminating whether riboflavin laurate could transport as the prototype.

Riboflavin laurate (RL) (Figure 1), designed as a new sustained-release and oil preparation of riboflavin, has been widely used as an army medication for riboflavin deficiency in various diseases for decades. In addition, current views merely consider that riboflavin, not free riboflavin laurate, is released and transported from intramuscular injection site to whole body, thereby producing sustained clinical effects (Figure 2). Until recent years, several research groups demonstrated undoubtedly that riboflavin laurate shown the potential to protect the patients from the oral or gastrointestinal mucositis induced by chemotherapy or radiotherapy in clinic [8–10]. Interestingly, either *via* oral or injection, riboflavin could not be perfectly capable. Based on clinical phenomenon, we assume that riboflavin laurate could play a stronger role in the prevention and treatment than riboflavin.

However, as little is known about the preventive effect at the cellular level, we investigated the possible role of riboflavin laurate in ameliorating chemotherapy or radiotherapy induced toxicities *in vitro*. In addition, to determine whether riboflavin laurate can release and transport across the cell membrane as the free type, we established the human colon carcinoma cell line (Caco-2) model to screen the transport properties of the promising drugs [11,12], ultimately, to illuminate what the leading contributor to oral or gastrointestinal mucositis is.

## 2. Results and Discussion

### 2.1. The Protective Properties of Chemotherapy, Radiotherapy-Induced Toxicity on Helf Cells

**Figure 3:**
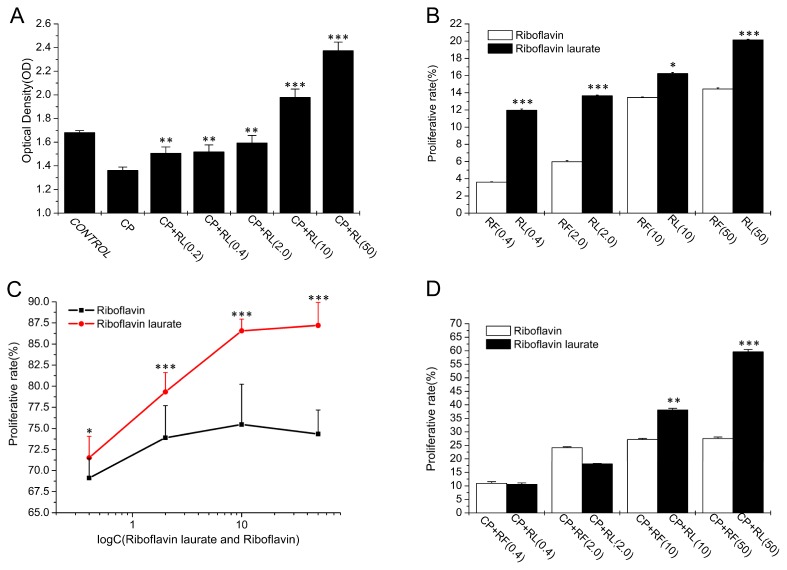
The protective and therapeutic properties of chemotherapy-induced toxicity on Helf cells. (**A**) Riboflavin laurate (0.2, 0.4, 2, 10, 50 μmol/L ) exhibit the high protective properties on CP-induced Helf cells; (**B**) The effects of different concentrations of riboflavin laurate and riboflavin on the normal Helf cells; (**C**) The preventive effects of riboflavin laurate and riboflavin on the Helf cells induced by CP (5 μmol/L) for 5 h; (**D**) The preventive effects of riboflavin laurate and riboflavin on the Helf cells induced by CP (5 μmol/L) for 72 h.

In our results, riboflavin laurate (0.2, 0.4, 2, 10, 50 μmol/L) exhibited the high protective properties on cisplatin (CP) (5 μmol/L)-induced the human embryonic lung fibroblasts (Helf cells), and the effects were concentration dependent. Apart from this finding, to our surprise, we observed that the cells, exposed to the higher concentration of riboflavin laurate (10, 50 μmol/L), proliferated greater as compared with the control group (*p* < 0.001) (Figure 3A). To confirm the conclusion, a further experiment was designed to examine the proliferative implications of riboflavin laurate and riboflavin on the normal Helf cells, and our data indicated that either riboflavin laurate or riboflavin can promote the normal Helf cells to grow. Furthermore, riboflavin laurate had better proliferative effect than riboflavin (*p* < 0.05), and the proliferative rate of riboflavin laurate (50 μmol/L) was up to 20.14% (Figure 3B). In another study, the cells induced by CP (5 μmol/L) for 5 h, were incubated with the fresh medium containing riboflavin laurate or riboflavin (0.4, 2, 10, 50 μmol/L), and cellular viabilities were determined after 72 h. Compared with CP-induced group, we could know that riboflavin laurate and riboflavin also ameliorated CP-induced toxicities on Helf cells. Importantly, the protection of riboflavin laurate was greater than that of riboflavin (*p* < 0.05) (Figure 3C). Then the cells were incubated with the medium containing CP (5 μmol/L) and riboflavin laurate (0.4, 2, 10, 50 μmol/L) or riboflavin (0.4, 2, 10, 50 μmol/L) until 72 h. An interesting observation was readily apparent that riboflavin with low concentrations had a similar protective effect to riboflavin laurate, yet high concentrations of riboflavin laurate shown the better and stronger protection (*p* < 0.05) (Figure 3D).

The Helf cells under different ^6^°Co-γ radiative treatments (0Gy, 2Gy, 4Gy, 8Gy, 16Gy), caused major damage as evidenced by down-regulation of cellular viability in ^6^°Co-γ radiative treated groups with respect to the control group (0Gy). After the exposure of different radiative dosages of ^6^°Co-γ, the cells were incubated in medium containing riboflavin laurate or riboflavin (20, 50, 100 nmol/L).

**Figure 4:**
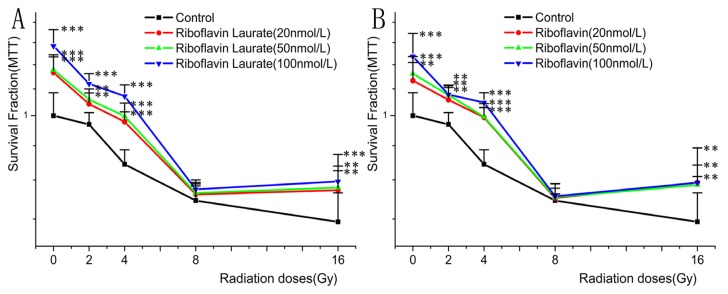
The protective and therapeutic properties of radiotherapy-induced toxicity on Helf cells. (**A**) The effects of riboflavin laurate on cell exposed by different radiative dosages of ^6^°Co-γ; (**B**) The effects of riboflavin on cell exposed by different radiative dosages of ^6^°Co-γ.

From these results, it not only further confirmed that either riboflavin laurate or riboflavin could promote the growth of normal Helf cells, also decreased the damage of ^6^°Co-γ radiations (*p* < 0.05), and the both effects were concentration dependent (Figure 4A,B). Additionally, when the supplements were in low concentration, riboflavin contained the same protective effect as riboflavin laurate. However, the DRF of riboflavin laurate (100 nmol/L) was 3.124 and was larger than that of riboflavin (Table 1), indicating the effect of riboflavin laurate in ameliorating radiotherapy-induced toxicities was greater as compared with that of riboflavin.

### 2.2. Transport Characteristics of Riboflavin Laurate by Caco-2 Cell Monolayer

#### 2.2.1. Measurements of Caco-2 Cell Monolayer Integrity

After being seeded for 21 days, we could see the confluent monolayers of Caco-2 cells, using an inverted light microscope. Then every monolayer was assessed by means of TEER measurements, and all of the monolayers of TEER reached 500 Ω × cm^2^. At the same time, sodium fluorescein solutions (2.0 mg/mL) were added on the apical side and incubated for 60 min, and the samples were detected by Multilabel Plate Reader. The Papp of sodium fluorescein was (0.284 ± 0.042) × 10^−6^ < 0.5 × 10^−6^ (cm/s).

In summary, all of the results indicated that Caco-2 cell monolayers were established. Subsequently, transport experiments could be performed.

#### 2.2.2. Transport across Caco-2 Cell Monolayer

**Figure 5:**
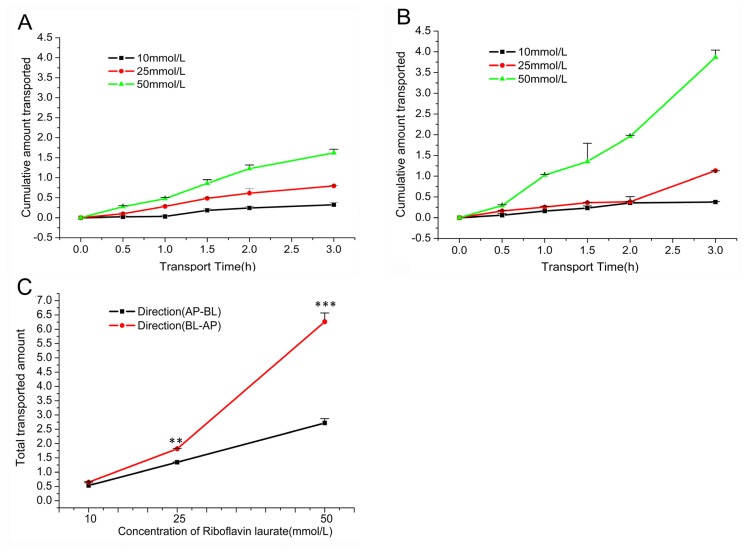
The influence of transport directions and concentrations of riboflavin laurate on transport across the Caco-2 cell monolayer. (**A**) The influence of AP-BL of riboflavin laurate (10, 25, 50 mmol/L) on cumulative amount transported over time; (**B**) The influence of BL-AP of riboflavin laurate (10, 25, 50 mmol/L) on cumulative amount transported over time; (**C**) The total amount transported of riboflavin laurate (10, 25, 50 mmol/L) of transport directions.

Using the Caco-2 cell monolayer, we known that riboflavin laurate can transport as the prototype, and the transport was linear with time and concentration-dependent. In addition, the cumulative amount transported of riboflavin laurate (10 mmol/L, 25 mmol/L, 50 mmol/L) in different directions (AP-BL and BL-AP) was shown in Figure 5A and B, respectively. By comparison, we found that the total transported amount of riboflavin laurate in the apical to basolateral was significantly lower than that of transport in the basolateral to apical (*p* < 0.05) (Figure 5C and Table 2). From the results of Table 2, the Papp of riboflavin laurate (10, 25, 50 mmol/L) in the apical to the basolateral direction (Papp (AP-BL)) was (4.417~4.495) × 10^−6^ cm/s and percent transport (%T) was between 5.34 and 5.44, indicating riboflavin laurate had a substantial transport across the cell membrane. On the other hand, on account of the result that Papp (AP-BL) was lower than the Papp (BL-AP) (ranged from 5.357 × 10^−6^ cm/s to 10.35 × 10^−6^ cm/s), their EfR of riboflavin laurate was between 1.213 and 2.303, manifesting the transport of riboflavin laurate may follow an efflux factor.

As is known to us, this is a frequent phenomenon in cancer patients receiving chemotherapy or radiotherapy, many of whom develop oral or gastrointestinal mucositis and suffer from painful and deep mucosal ulcer [13,14]. Hence, amelioration or relieving the pain of oral or gastrointestinal mucositis is one of the important clinical challenges in the cancer therapy area [15].

In recent years, the importance of riboflavin laurate on nutrition, especially in cancer patients, has been largely appreciated. With the increasing investigations of riboflavin laurate, more and more evidences indicate that riboflavin laurate could prevent and therapy the oral or gastrointestinal mucositis induced by chemotherapy or radiotherapy, and the contribution may result from antioxidant activity of riboflavin laurate, which attenuates the chemotherapy or radiotherapy-induced increase in apoptotic indices, with a decrease in oxidative burden, increased Bcl-2/Bax, and improved functional and structural integrities.

Designed as a sustained-release preparation, riboflavin laurate produces sustained therapeutic and preventive effect of unrecognized deficiency of this micronutrient. Allegedly, the finding helps us address the problems that riboflavin can not be synthesized in the body, and an easily deficiency syndrome is recurrent.

In our study, we want to demonstrate the effects of riboflavin laurate in preventing oral or gastrointestinal mucositis induced by chemotherapy or radiotherapy at the cellular level, so the Helf cell is used, which can provide a useful model to study components with toxicity or antioxidant activity. To date, CP is a prominent and effective broad spectrum anticancer drug, which is widely used as a therapy for head and neck, breast, lung, prostate and cervix cancer. However, despite being an effective anticancer agent, CP is limited in the clinical usage by various side effects, for example, oral or gastrointestinal mucositis is one of the most important adverse reactions [16,17].

In addition, the Caco-2 cell monolayer is a well-established model for screening the transport, intestinal absorption and metabolism properties of new drugs [18]. Therefore, using the model, we first investigate whether riboflavin laurate could transport across Caco-2 cell monolayer as the prototype and illuminate the transport characteristics of riboflavin laurate, helping us clearly understand what the active ingredient ameliorating or relieving the pain of oral or gastrointestinal mucositis is.

Based on our results, riboflavin laurate indeed shows the protective and therapeutic effects on the chemotherapy or radiotherapy- induced Helf cells successfully. Intriguingly, the perfect influence is greater than that of riboflavin, particularly on the chemotherapy-induced toxicities. Fortunately, the findings are in accordance with previous clinical investigations that riboflavin laurate is mainly used to prevent cancer patients from oral ulcers after chemotherapy, and the oral riboflavin can not produce the prefect therapeutic effect.

**Figure 6:**
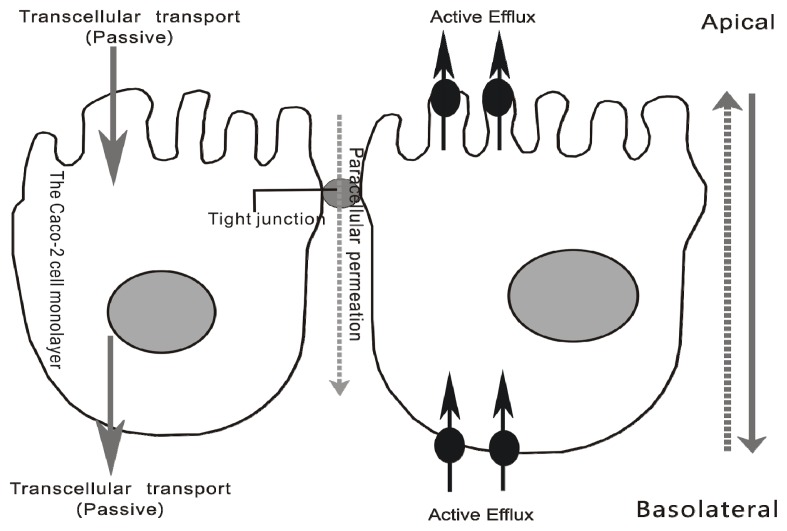
The transport mechanisms of riboflavin laurate across Caco-2 cell monolayer. Riboflavin laurate can transport as the prototype, and the transport mechanisms are complex, not only via passive transcellular transport, also through active efflux.

On the other hand, the data from transport experiments by Caco-2 cell monolayer, suggest that riboflavin laurate could release and transport across the cell membrane, not only *via* passive transcellular transport, but also through active efflux (Figure 6). Although the transport mechanisms are complex, it is clearly feasible and worth research attention, that riboflavin laurate can transport as the prototype, which may be the reason of the effects on oral mucositis induced by chemotherapy or radiotherapy.

## 3. Experimental Section

### 3.1. Materials

Helf cell and Caco-2 cell were obtained from Cell Resource Center, IBMS, PUMC. Riboflavin laurate and riboflavin were endowed by SHENZHEN SALUBRIS PHARMACEUTICALS CO., LTD. Sodium fluorescein and Thiazolyl Blue Tetrazolium Bromide (MTT) were purchased from Sigma company. Millicell cell culture inserts (MA, 01812) and Millicell ERS were from Millipore Corporation, USA. Cell culture medium and reagents were from Gibco Laboratories. HPLC (L-7000) was from Hitachi, Japan. Multiskan Ascent Plate Reader was bought from Thermo Scientific, Finland. ^6^°Co-γ facility was provided by Beijing Institute of Radiation Medicine. Multilabel Plate Reader 2030 was purchased from PerkinElmer, USA.

### 3.2. Methods

#### 3.2.1. Riboflavin Laurate Ameliorates Chemotherapy, Radiotherapy-Induced Toxicities

In this study, Helf cells were seeded into 96 well plates at an appropriate density (3000 cells per well) in 100 μL DMEM containing 10% FBS. And the culture plates were kept in a 37 °C humidified atmosphere containing 95% air and 5% CO_2_. The next day, the cells were treated with the following combinations: CP group, riboflavin laurate–CP group, riboflavin–CP group and control group. Before the incubation period with different concentrations of riboflavin laurate or riboflavin, CP (5 μmol/L) was supplemented into three former groups for 5 h, then the medium was removed and the cells were incubated in 200 μL DMEM medium containing riboflavin laurate (0.4, 2, 10, 50 μmol/L) or riboflavin (0.4, 2, 10, 50 μmol/L), and CP group was replaced by the same volume of blank DMEM medium. The control group received no treatment. After 72 h, the medium was removed and the 100 μL MTT solution (0.5 mg/mL in medium) was added to the culture plates. Then, incubating for 4h at 37 °C, the MTT reaction medium was removed away and formazan blue was solubilized by 200 μl of DMSO. Finally, values of absorbance were detected by Multiskan Ascent at 570 nm [19–21].

According to the above method, Helf cells were planted onto five culture plates. The cells of five culture plates were exposed to different ^6^°Co-γ radiative treatments: 0Gy, 2Gy, 4Gy, 8Gy, 16Gy. For our purpose, the cells of every culture plate were divided into seven groups: control group and three groups of riboflavin laurate (20, 50, 100 nmol/L) and three groups of riboflavin (20, 50,100 nmol/L). Before incubated in medium containing riboflavin laurate or riboflavin, cells were exposed by different radiative dosages of ^6^°Co-γ. After the exposure period, cellular viability was determined by MTT test. The cellular viability of control group (0Gy) served as survival fraction 1, then the survival fractions and the linear equations would be determined, “*y*” is the survival fraction and “*x*” is dose of radiation. Then the dose reduction factor (DRF) was calculated by the following formula:

$$\text{DRF}={\text{D}}_{90}\;\text{of riboflavin laurate or riboflavin group}/{\text{D}}_{90}\;\text{of control group}.$$

#### 3.2.2. Transport Studies Performed by Caco-2 cell Monolayer

Caco-2 cells were seeded onto Millicell cell culture inserts (1.12 cm^2^ surface area, 0.4 μm pore size) in 12-well plates at a seeding density of 1 × 10^5^ cells/cm^2^. MEM Alpha Modification Medium, supplemented with 10% fetal bovine serum, 1% L-Glutamine as well as with 1% nonessential amino acids, was used as culture medium. Cell cultures were kept at 37 °C in an atmosphere of 95% air and 5% CO_2_. Cell medium was changed every day until 21 days [22,23].

Prior to experiments, the integrity of the monolayer was ensured by an inverted light microscope and transepithelial electric resistance (TEER) measurements. In addition, the permeability of sodium fluorescein (marker for barrier integrity) was measured before the transport experiments [24].

Transport experiments were performed as described previously [25], using PBS buffer (pH 7.4, 37 °C) as the transport medium. In our research, to study the apical to basolateral transport, 1.5 mL of the medium was added to the basolateral chamber (receptor) of the Millicell cell culture inserts and then 0.5 mL of the transport medium with riboflavin laurate (10, 25, 50 mmol/L) was added to the apical side (donor). By contrast, the basolateral to apical transport was initiated with 1.5 mL of the transport medium containing riboflavin laurate (10, 25, 50 mmol/L) in the basolateral side (donor), and 0.5 mL of the medium in the apical side (receptor). Thereafter, samples (100 μL) were taken out from the receiver side at 0.5, 1.0, 1.5, 2.0 and 3.0 h, and the equivalent volume of fresh transport medium was supplemented. Finally, all the samples were analyzed by HPLC system [26,27], and free riboflavin laurate was measured by the method developed in our lab.

Chromatographic separation was performed on a C18 column (ODS250, DIKMA). The mobile phase A (Solvent A) was water, while the mobile phase B (Solvent B) was methanol. The elution profile (1.0 mL/min) was as follows: 0–1 min, isocratic elution, 80% solvent A/ 20% solvent B; 1–6 min, linear gradient from 80% solvent A/ 20% solvent B to 40% solvent A/ 60% solvent B; 6–10 min, linear gradient elution, from 40% solvent A/ 60% solvent B to 30% solvent A/ 70% solvent B; 10–15 min, mobile phase restore to 80% solvent A/ 20% solvent B. UV-VIS detection wavelength was 375 nm, and the whole HPLC system was stable at 30 °C.

In our experiment, the standard curve equation was Y = 2903.4X + 175.99, R^2^ = 0.9951. Additionally, the experimental method provided suitable precision, accuracy and limit of detection (LOD). Therefore, the HPLC-UV system was a creditable method for rapid analysis of riboflavin laurate.

Then, the apparent permeability coefficient (Papp), percent transport (%T) and the efflux ratio (EfR) were determined [28]. Papp, %T and EfR were calculated as follows:

$$\begin{array}{c} \text{Papp}=\text{dQ}/(\text{dt}\times \text{A}\times {\text{C}}_{0})\\ \%\text{T}=\text{C}(\text{Receptor})/\text{C}(\text{Donor})\\ \text{EfR}=\text{Papp }(\text{BL}-\text{AP})/\text{Papp }(\text{AP}-\text{BL}) \end{array}$$

Where, dQ/dt is the rate of appearance of riboflavin laurate on the receptor side; C_0_ is the test compound initial concentration on the donor side; A is the surface area of the Millicell cell culture inserts; C(Receptor) is cumulative concentration on the receptor side; C(Donor) is initial concentration on the donor side.

### 3.3. Statistics

Data were expressed as mean ± SD. The t-test was used for statistical analysis and statistical significance was defined (* *p* < 0.05; ** *p* < 0.01; *** *p* < 0.001).

## 4. Conclusions

Riboflavin laurate promises to have potential in the protection of patients from the oral or gastrointestinal mucositis induced by chemotherapy or radiotherapy at the cellular level. In addition, the protective effects may be produced by the prototype of riboflavin laurate, rather than simply by the released riboflavin.

In conclusion, we believe the promising drug, riboflavin laurate, will be widely needed by cancer patients receiving chemotherapy or radiotherapy, especially as many of them have developed mucositis. However, further researches should be considered to aim at investigating the therapeutic mechanisms of riboflavin laurate on oral or gastrointestinal mucositis, and in describing the definite transport characteristics of the promising drug.

## Figures and Tables

**Table 1:** Determinations of dose reduction factor (DRF) on Helf cells treated with riboflavin or riboflavin laurate.

Group	y = a*x* + b	D_90_ (Gy)	DRF
Control	y = −0.0218*x* + 0.965	2.982	1.000
Riboflavin (20 nmol/L)	y = −0.0249*x* + 1.090	7.631	2.559
Riboflavin (50 nmol/L)	y = −0.0271*x* + 1.112	7.823	2.624
Riboflavin (100 nmol/L)	y = −0.0305*x* + 1.163	8.623	2.892
Riboflavin laurate (20 nmol/L)	y = −0.0270*x* + 1.100	7.407	2.484
Riboflavin laurate (50 nmol/L)	y = −0.0276*x* + 1.116	7.826	2.625
Riboflavin laurate (100 nmol/L)	y = −0.0335*x* + 1.212	9.313	3.124

**Table 2:** The coefficients (Papp, %T, and EfR) of different concentrations of riboflavin laurate transport across the Caco-2 cell monolayer.

Concentration	AP-BL		BL-AP		EfR

	Papp (10^−6^, cm/s)	%T	Papp (10^−6^, cm/s)	%T	
10(mmol/L)	4.4173 ± 0.7591	5.34 ± 0.92	5.357 ± 0.2442	6.48 ± 0.30	1.213
25(mmol/L)	4.4431 ± 0.0003	5.37 ± 0.06	6.004 ± 0.0440	7.26 ± 0.05	1.351
50(mmol/L)	4.4947 ± 0.2530	5.44 ± 0.31	10.35 ± 0.5057	12.52 ± 0.61	2.303
